# Synthesis and characterization of tannic acid–PEG hydrogel *via* Mitsunobu polymerization†

**DOI:** 10.1039/c9ra09229c

**Published:** 2020-01-09

**Authors:** Chen Chen, Xi-wen Geng, Ya-hui Pan, Yu-ning Ma, Yu-xia Ma, Shu-zhong Gao, Xiao-jun Huang

**Affiliations:** Key Laboratory of New Material Research Institute, Department of Acupuncture-Moxibustion and Tuina, Shandong University of Traditional Chinese Medicine Jinan 250355 China myning0405@163.com myxia1976@163.com; Key Laboratory of Stem Cell and Translational TCM, Experimental Center, Shandong University of Traditional Chinese Medicine Jinan 250355 China; MOE Key Laboratory of Macromolecular Synthesis and Functionalization, Department of Polymer Science and Engineering, Zhejiang University Hangzhou 310027 China

## Abstract

Tannic acid (TA) based materials have received significant interest owing to their broad spectrum of chemical and biological properties. Herein, a novel tannic acid based hydrogel, TA–PEG hydrogel, was synthesized *via* Mitsunobu polymerization/polycondensation, in which TA and polyethylene glycol (PEG) were simply crosslinked together by ether linkages. This method was performed in one pot, straightforward, metal free and robust, ignoring the strong ionic/hydrophobic interactions between tannic acid and PEG. Bearing catechol and pyrogallol units from TA, TA–PEG hydrogel did not only reduce the silver and gold precursor, but also served as a capping agent and stabilizer for the *in situ* formed Au and Ag nanoparticles (NPs). Furthermore, the antioxidant activity of the hydrogel was excellent (94%) in the case of 1,1-diphenyl-2-picrylhydrazyl (DPPH) radical scavenging. TA–PEG hydrogel also showed antibacterial activity against *Staphylococcus aureus* and *Escherichia coli*. This work suggested a new method leading to polyphenol based soft materials rather than a complex coacervated microstructure. The resulting TA–PEG hydrogel has potential application in biomedical materials.

## Abstract 2

Mitsunobu polymerization was involved to construct bulk hydrogel *via* direct PEGylation of tannic-acid under mild condition.

## Graphical abstract

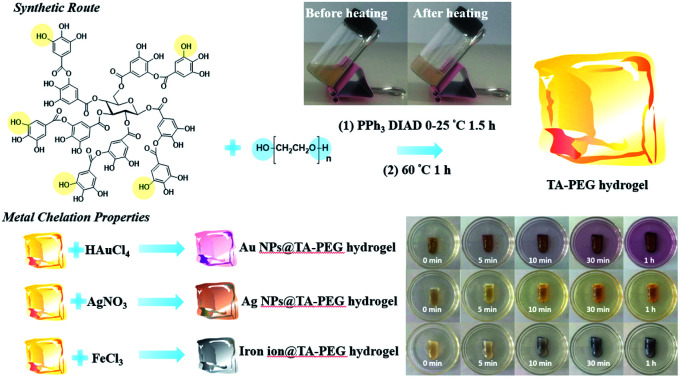

## 1. Introduction

The Mitsunobu reaction, essentially a nucleophilic reaction, is the dehydrative coupling of a primary or secondary alcohol to an acidic pronucleophile (NuH) mediated by a redox combination of a trialkyl or triarylphosphine and a dialkyl azodicarboxylate. Named after its discoverer Professor Oyo Mitsunobu in 1967, this reaction has enjoyed a privileged role in organic synthesis and medicinal chemistry because of its scope, stereospecificity, and mild reaction conditions.^1,2^ Apart from esters, a wide range of compounds that include amines, azides, ethers, cyanides, thiocyanides, thioesters, and thioethers can be synthesized using a Mitsunobu protocol.^3^ Though being prevalent in organic synthesis and medicinal chemistry, this reaction results in the formation of a full equivalent of triphenylphosphine oxide, which is recognized as lack of atom economy. Meanwhile, similarity in polarity among unreacted reagents, byproducts and the final small molecule product very often requires tedious column chromatography.^4^ Efforts has been made to conquer the drawbacks of Mitsunobu reaction, separation tags for facilitating purification of reaction mixtures have gained considerable momentum in the recent years.^5^ Several modified Mitsunobu reagents allowed ready separation of the product after a phase-switching reaction.^6^ However, an additional reaction or processing step is needed to remove the phase tag and bring the coupled product back to the desired organic/liquid phase. Other modified methods including the employment of polymer-supported reagents, basic phosphines and fluorous reagents were cost enhancing and, of most significance, inevitable from preparative column chromatography.^7–9^ Consequently, Mitsunobu reaction is mainly employed either as an intermediate step for the synthesis of functional organic small molecules, or as a post-polymerization functionalization route.^10–14^ Illuminated by the work of Chong-Bok Yoon and Hong-Ku Shim^15,16^ in which polyimides were obtained *via* Mitsunobu polycondensation, it is believed that Mitsunobu polymerization or Mitsunobu polycondensation is a potential method to simplify the separation process as well as a promising way leading to a bunch of brand-new polymers due to its wide scope. Rather than synthesis of linear polyimides, crosslinked bulk polymers obtained *via* Mitsunobu polymerization are promising since the properties of nucleophiles (carboxylic acids, phenols, thiols, imines, purine/pyrimidine bases and so on) and substrates can be integrated together without tedious column chromatography separation process.

Phenols, normally p*K*_a_ < 11, can act as nucleophiles in the Mitsunobu coupling.^17^ Generally, phenolic compounds are naturally occurring materials widely found in living organisms.^18^ The significant interest in phenolic compounds in recent years is attributed to their broad spectrum of chemical and biological properties. Plant phenols such as tannic acid (TA), green tea catechins and dietary flavonoids are also capable of forming universal coatings, and they have superiorities over dopamine in their abundant natural resources and nontoxicity.^19^ TA is of enhancing interests for researchers nowadays owing to its ability to complex or crosslink macromolecules at multibinding sites through hydrogen bonding, ionic bonding and hydrophobic interactions.^20–22^ The coordination-triggered deposition of TA–metal films on a wide range of substrates, which has been recognized as a robust and diverse method leading to organic–inorganic hybrid materials with synergistic properties to meet complex biological requirements.^23^ Therefore, TA is an ideal crosslinker for hydrogel formation.

Although TA–macromolecule complexes have been studied for decades, straightforward crosslinking polymer solution into hydrogel is still a challenge because the strong ionic/hydrophobic interactions often give rise to coacervation rather than network formation.^24^ Recently, Fan *et al.*^25^ reported a simple route to crosslink commercially available polymers such as polyvinylpyrrolidone (PVP), PEG, poly(sodium 4-styrenesulfonate) (PSS), and poly(dimethyldiallylammonium chloride) (PDDA) into supramolecular hydrogels by taking advantage of diverse interaction modes of tannic acid. Other than the above supramolecular method, herein we proposed a covalent-binding route in which tannic acid was subjected to direct PEGylation *via* Mitsunobu polymerization. The polymerization was performed in a mild condition (0–25 °C) in presence of chemical available PPh_3_/diisopropyl azodicarboxylate (DIAD). Since the final product was a light-coloured bulk hydrogel, unreacted reagents and byproducts (triphenylphosphine oxide) could be removed by successive washing using alcohol and water, alternatively. The resulted TA–PEG hydrogel could not only act as macromolecular substrates for Fe^3+^ chelation, but also had the ability to generate Au and Ag nanoparticles *in situ* rapidly within 5–10 min owing to the reducibility originated form TA. Antibacterial assay manifested that TA–PEG showed desirable antibacterial activity against *Staphylococcus aureus* (*S. aureus*) and *Escherichia coli* (*E. coli*). This hydrogel has the potential for applications in wound dressing, tissue engineering and drug loading.

## 2. Experimental section

### 2.1. Materials

Tannic acid (TA), PPh_3_, diisopropyl azodicarboxylate (DIAD), CH_3_CN were purchased from Aladdin Co., Shanghai, China. PEG 6000 (mean average MW = 6000) was purchased from Ourchem Co., Shanghai, China. All other reagents were commercial chemicals and used as received except specially claimed.

### 2.2. Synthesis of TA–PEG hydrogel

TA–PEG hydrogel was synthesized by a two-step method *via* a classical Mitsunobu condensation route. Typically, to a 50 mL polypropylene centrifuge tube, PEG 6000 (1.5 g, 0.25 mmol) and PPh_3_ (660 mg, 2.52 mmol) were added and then dissolved by 15 mL CH_3_CN. Subsequently, TA (170 mg, 0.10 mmol) in 5 mL was poured into the above mixture to obtain a milky white solution. DIAD (0.48 mL, 2.52 mmol) in a 1 mL syringe was carefully added drop by drop under vigorous stirring at 0 °C. At the end of this titration, the color of the mixture turned to deep orange color. Then, the mixture was gradually heated to 25 °C under stirring for 1.5 h to obtain a light yellow viscous solution. For gelation process, the above mixture was poured into polypropylene molds, sealed and heated to 60 °C for 1 h. Finally, a light yellow transparent TA–PEG hydrogel was obtained, which was washed successively by ethanol and water for 3 times. The resulted hydrogel was lyophilized at −50 °C for further uses.

### 2.3. Equilibrium swelling measurement of TA–PEG hydrogel

The freeze-dried hydrogel of known weight was immersed in distilled water at room temperature until equilibrium then the excess water on the samples surface were absorbed with filter paper and the swollen hydrogels were weighed. The water uptake was calculated as follows:
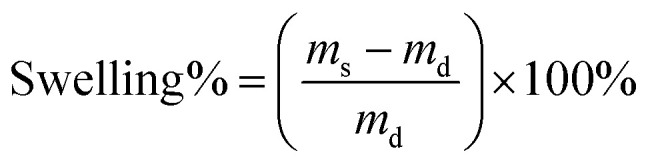
where *m*_d_ and *m*_s_ are the weight of the initial weight of dried hydrogel and the weight of the swollen hydrogel.

### 2.4. Dynamic rheology studies

Rheology measurements were carried out on the as-prepared TA–PEG hydrogel with a stress-controlled rheometer MCR302 (Anton Paar) equipped with parallel plate geometry (diameter, 25 mm, gap of 1 mm). Silicone oil was laid on the edge of the fixture plates to prevent solvent evaporation. The *G*′ (storage modulus) and *G*′′ (loss modulus) in function of strain (1–200%) were measured, respectively, at 1 Hz frequency.

### 2.5. *In situ* formation of Au and Ag NPs by TA–PEG hydrogel

Hydrogels in volume of 2 mL were used for *in situ* formation of Au and Ag nanoparticles. As to the formation of Au NPs, the swelling hydrogel was directly added to HAuCl_4_ aqueous solution (0.01 wt%, 10 mL) under constant shaking at 50 rpm for 1 h. Similarly, AgNO_3_ aqueous solution (0.1 wt%, 10 mL) was utilized to form Ag NPs.

### 2.6. Antioxidant activity of TA–PEG hydrogel

The antioxidant activity of the hydrogel was measured with DPPH radicals (DPPH˙) using a standard method.^26^ DPPH˙ is a well-known radical and a trap (“scavenger”) of other radicals. Therefore, the rate reduction of a chemical reaction upon the addition of DPPH˙ was used as an indicator of the radical nature of that reaction. The evaluation was conducted as follows; an amount of 500 mg of dried hydrogel was immersed in a 50 mL centrifugal tube containing 30 mL of 0.15 mM DPPH/methanol solution. The absorbance at 517 nm was measured using a UV-vis spectrophotometer after the solutions had been allowed to stand in the dark for 30 min. Lower absorbance of the solution indicates higher DPPH-scavenging activity. The DPPH-scavenging activity was calculated using the following formula:
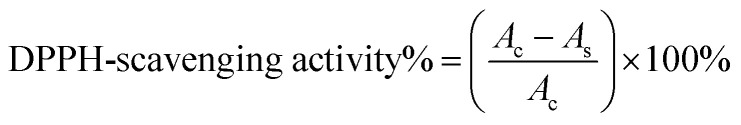


In the equation, *A*_s_ and *A*_c_ are the absorbance of the sample and of the control at 517 nm, respectively.

### 2.7. Antibacterial assay

Two kinds of bacteria were used as test microorganisms in this study. Test bacteria included *Staphylococcus aureus* (RCMB 000106) (as Gram positive bacteria) and *Escherichia coli* (RCMB 000107) (as Gram negative bacteria). The strains were stored at 4 °C. The medium used for growing bacteria was universal nutrient agar. Antibacterial activity was determined by the agar well-diffusion method. Briefly, agar plates were seeded with test microorganisms and kept for 30 min until the medium solidified. Circular pieces of approximately 1 cm in diameter of the test hydrogels were added. Plates were kept for 12 h at 4 °C prior to incubation at the appropriate temperature for the bacteria. Plates were examined on a daily basis to check for the development of growth inhibition zones around the loaded hydrogels.

Antimicrobial activity against *Escherichia coli* was also carried out *via* a shake flask test.^27^ TA–PEG hydrogel and Ag NPs@TA–PEG hydrogel (1.20 ± 0.05 g, 2 mL) were sterilized with UV light for 30 min and subjected to 103 kPa for 15 min in a high-pressure steam sterilizer. A typical single colony for *Escherichia coli* that had been cultured for 24 h was inoculated into nutrient broth and cultured for 18 h at 37 ± 2 °C to prepare an inoculum suspension. The inoculum suspension was diluted with PBS solution to approximately 108 colony forming units (CFU) mL^−1^, which was measured with a spectrophotometer. Each tested sample was added into a 250 mL conical flask with 70 mL of 0.03 mol L^−1^ PBS solution, and then 1 mL of bacterial suspension was added. The flask was finally plugged with a stopper. Next, the conical flask was shaken and cultured at 200 rpm for 18 h at 37 °C. When the incubation time was reached, 1.0 mL of the sample suspension was taken from each flask and transferred to 9.0 mL of 0.03 mol L^−1^ PBS solution in a test tube and was thoroughly mixed. Serial dilutions were made with a 10-fold dilution method. Then, 1.0 mL of each sample with a certain dilution factor was transferred to a nutrient agar dish. After 24 h of incubation at 37 °C, the CFU number in the dish was counted. If the CFU number in the dish with the smallest dilution factor was <30, then the actual number was recorded. If there was no CFU, it was recorded as 0. The total viable bacteria concentration in each conical flask was determined by multiplying the CFU number by the dilution factor. Then the bacteriostasis rate of each tested sample was calculated by equation:
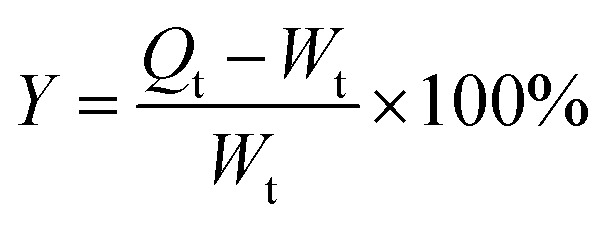
where *Y* is the bacteriostasis rate, *W*_t_ is the concentration of bacteria colonies for the control sample (CFU mL^−1^) and *Q*_t_ is the concentration of bacterial colonies for the test sample (CFU mL^−1^).

This antibacterial experiment was repeated for three times for each sample. In theory, as the amount of antimicrobial agent increased, the number of colonies surviving on the nutrient agar dishes should gradually decrease. Therefore, the last nutrient agar dish with surviving colonies was selected and the number of colonies was counted. The test with the highest number of colonies of these three tests was reported.

### 2.8. Other characterization

FTIR spectra were recorded using a Bruker Vector 22 Fourier Transform Infrared Spectrometer, with the samples pressed into potassium bromide pellets. TA–PEG hydrogel in volume of 2 mL were immersed in 10 mM PBS (pH 7.4) and incubated in air bath under 50 rpm shaking at 37 °C for 10 days. Aliquot samples were taken out and lyophilized for Nuclear Magnetic Resonance (NMR) measurements. ^1^H NMR and ^13^C NMR measurements (a Varian Gemini-300 spectrometer operated at 600 MHz) were carried out to evaluate the chemical structure of the remaining hydrolyzed polymer dissolved in DMSO-*d*_6_. The morphology of TA–PEG hydrogel were observed by Scanning Electron Microscopy (SEM) with a Hitachi S4800 SEM at an acceleration voltage of 20.0 kV. Transmission Electron Microscopy (JEM-1200EX, NEC, Japan) was used to observe the nanostructures of Au and Ag at an accelerating voltage of 120 kV.

## 3. Results and discussion

### 3.1. Synthesis of TA–PEG hydrogel

**Scheme 1:**
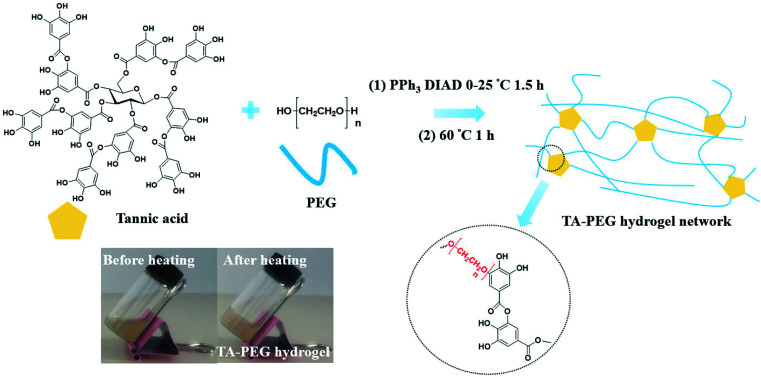
Synthetic route leading to TA–PEG hydrogel *via* Mitsunobu polymerization.

As illustrated in Scheme 1, TA–PEG hydrogel was synthesized by a two-step Mitsunobu polymerization. Tannic acid, with a p*K*_a_ of 10 approximately, could act as a acidic nucleophile in the PPh_3_–DIAD system. Considering the Mitsunobu reaction is essentially the dehydrative coupling of a primary or secondary alcohol, PEG without further chemical modification was chosen as the substrate. Furthermore, the hydrophilicity and amorphous nature of the PEG chain enabled the gelation *via* crosslinking. Dehydration occurred between the phenolic hydroxyl group of TA and the terminal hydroxyl group of PEG. We hypothesized that TA participates in the crosslinking process only with the outer pyrogallol groups due to the sterical hindrance that occurs. Further exploration proved that crosslinking occurred when the pyrogallol groups and hydroxyl groups were in equimolar quantities as can be seen in Table S1 (ESI†). As a result, covalent ether linkages were formed. The reaction phenomenon could be seen in Fig. S1 (ESI†), though PEG and TA were not compatible with each other in CH_3_CN owing to hydrogen bonding, the mixture gradually became homogeneous accompanied by viscosity increase as the reaction proceeded. In view of the low nucleophilicity of TA, the final gelation process required high temperature, which was moldable in glass bottles or generally used centrifugal tubes.

**Fig. 1:**
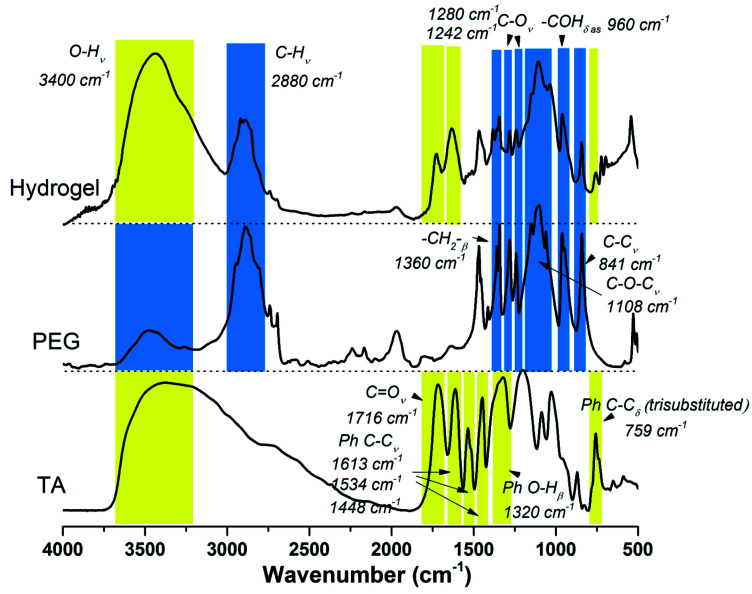
FT-IR spectrum of TA, PEG and TA–PEG hydrogel.

The chemical structures of the resulted hydrogel were further characterized by FI-IR spectroscopy. As demonstrated by Fig. 1, the new intensive absorption adsorption at 2880 wavenumber (cm^−1^) was attributed to the presence of C–H stretching upon hydrogel formation. Other representative peaks of PEG segment could be observed at 1280, 1242, 1108, 960 and 841 cm^−1^, which were assignable to C–O stretching, aliphatic C–C stretching, –COH scissoring and C–O–C stretching, respectively (the blue region in Fig. 1). Tannic acid segment showed some strong bands typical of polyphenols assigned to C=O stretching at 1716 cm^−1^, benzyl stretching at 1613, 1534 and 1448 cm^−1^, and trisubstituted benzene rings scissoring at 759 cm^−1^ (the yellow region in Fig. 1). Relative to the spectrum of PEG, strong wide peaks for hydroxyl group in hydrogel gained more intensive at 3400 cm^−1^, which indicated the introduction of residual phenolic hydroxyl group derived from TA. The NMR results were shown in Fig. S2 (ESI†). As to ^1^H NMR spectrum (Fig. S2a†), the single peak at 3.60 ppm was belonging to CH_2_CH_2_O group, the multiple peaks at 7.55 ppm and 7.62 ppm were assignable to aromatic protons. As to ^13^C NMR spectrum (Fig. S2b†), the strong single peak at 70 ppm typical of PEG backbone was assigned to (CH_2_CH_2_O)_*n*_. Aromatic carbons were also detected at 130–133 ppm.

**Fig. 2:**
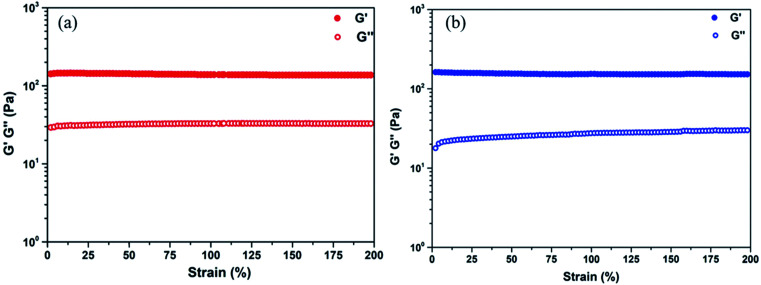
Strain sweep of the TA–PEG hydrogel saturated with water (a) and ethanol (b) at 1 Hz frequency at room temperature.

To investigate the internal morphology of the resulted TA–PEG hydrogel, the freeze dried sample were observed by SEM. As demonstrated by Fig. S3a and b (ESI†), one can find the internal and interior microstructure of the hydrogel. It can be seen that the hydrogel have a good three-dimensional network structure with irregular pores. The pore diameter of the hydrogel is about 10–50 μm, distributed randomly on the surface of the sample. The presence of pores play an important role in hydrogel properties such as compressibility, swelling, cell adhesion and nutrient diffusion.^28^ Hydrogel is normally recognized as a kind of 3D polymeric network that can swell in water. From the viewpoint of the synthetic process, this cross-linked gel seemed to be organogel since organic solvent was used to stabilize TA–PEG oligomer. However, this PEGylated gel was essentially hydrophilic and had the ability to swell under aqueous condition. So herein TA–PEG hydrogel was denoted as hydrogel. As illustrated by Fig. S3c (ESI†), the freeze-dried hydrogel was immersed in excess water at ambient temperature until equilibrium. TA–PEG hydrogel showed a swelling percentage at the range from 3000% to 3300%, due to the fact that tannic acid and PEO are hydrophilic molecules which contain a large amount of hydrophilic groups. Moreover, TA–PEG hydrogel also showed a swelling percentage of 1000% after 24 h immersion in ethanol. The result in Fig. 2 revealed that *G*′ was relatively independent over the entire strain range. Take water and ethanol as absorbing solvent for comparison, the *G*′ values were quite similar around 140–150 Pa. Obviously, *G*′ was significantly higher than *G*′′ for all hydrogels, confirming the existence of crosslinked network structure between tannic acid and PEG segments.

### 3.2. *In situ* formation of Au NPs and Ag NPs

**Fig. 3:**
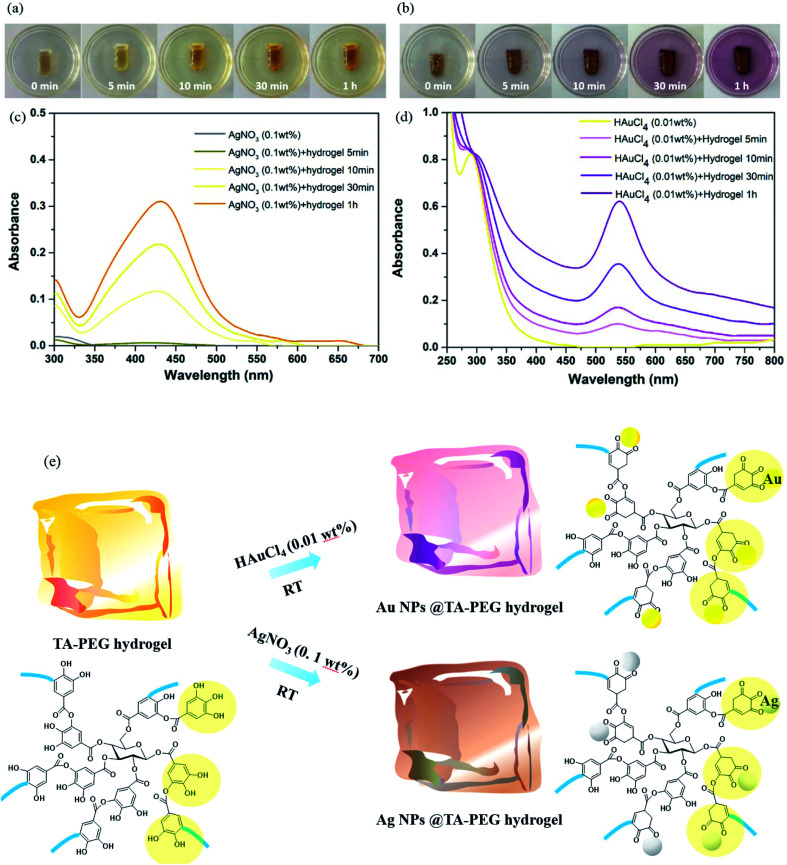
The *in situ* formation of Au&Ag NPs by using TA–PEG hydrogel as an reductant. (a) Photographs of TA–PEG hydrogel in treatment of AgNO_3_ (0.1 wt%); (b) photographs of TA–PEG hydrogel in treatment of HAuCl_4_ (0.01 wt%); (c) *in situ* formation of Ag NPs objected by UV-vis spectrum; (d) *in situ* formation of Au NPs objected by UV-vis spectrum; (e) schematic process for the Au or Ag NPs decorated TA–PEG hydrogel.

TA is a natural reducing agent extracted from plant sources, which is able to chelate with some metal ions and reduce them to metallic NPs.^29,30^ Since the mole ratio between the phenolic hydroxyl group and the hydroxyl group was set as 5 : 1, TA–PEG hydrogel inherited catechol and pyrogallol units from TA, readily for *in situ* reductions. As can be seen in Fig. 3a, TA did not only reduced silver and gold precursor, avoiding the usage of additional reducing agents or toxic reagents, but also served as capping agent and stabilizer for the *in situ* formed Au and Ag NPs.^31,32^ The corresponding reduction processes could be seen in Movies S1 and S2 (ESI†). As to the formation of Ag NPs, the gel color changed to brown as Ag^+^ diffused into it within 5 min in absence of heating. In addition to the color change, UV-vis (ultraviolet and visible) spectrum showed that an instinct peak appeared at 425 nm in 10 min (Fig. 3c), which probably corresponded to spherical silver nanoparticles. Accompanied by obvious tailing effect, this peak broadened and slightly shifted to longer wavelengths as the reduction time prolonged, which revealed the formation of larger Ag NPs. It was hypothesized that silver ions are first coordinated with the catechol and pyrogallol residues originated from TA, which are then *in situ* reduced to zerovalent silver with the redox reactions of phenol to form quinones and donate electrons.^33^ Similarly, Au NPs formed rapidly since the gel color changed to crimson as Au^3+^ diffused into the hydrogel within 5 min (Fig. 3b).^34^ UV-vis spectrum showed that the Au NPs displayed a strong absorption band at approximately 540 nm corresponding to its surface plasmon resonance (SPR) band (Fig. 3d). As the reduction process went by, violet stable gels were formed, suggested that the TA–PEG hydrogel reduced Au^3+^ ions and capped Au NPs *in situ* spontaneously within the polymeric network. Above all, the whole reduction reactions were proceeded in an aqueous solution at ambient conditions, which is compatible with green chemistry principles. Fig. 3e presents a schematic diagram to illustrate the *in situ* formation of the Ag or Au NPs decorated TA–PEG hydrogel (Ag NPs@TA–PEG hydrogel and Au NPs@TA–PEG hydrogel) using catechol and pyrogallol residues as the reducing agent and stabilizing agent.

**Fig. 4:**
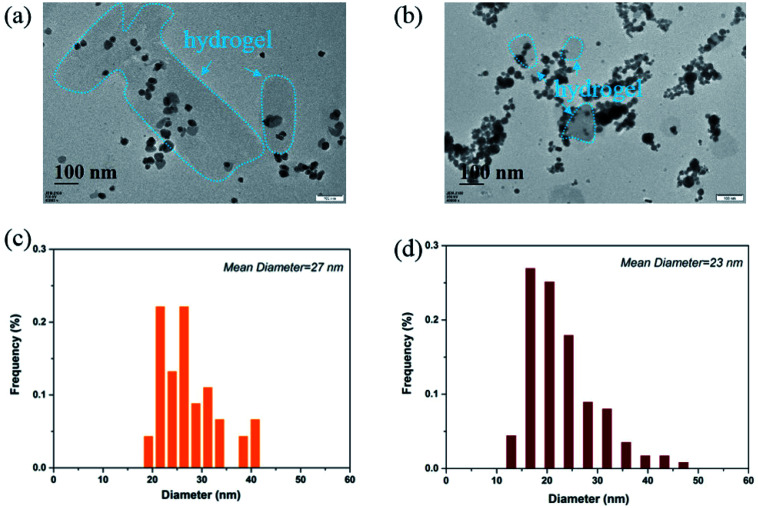
(a) The morphology of Ag NPs viewed by TEM; (b) the morphology of Au NPs viewed by TEM; (c) size distribution of Ag NPs; (d) size distribution of Au NPs.

It can be clearly seen from Fig. 4a that the Ag NPs are generally spherical in shape viewed by TEM. The resulted nanoparticles were further characterized by dynamic light scattering (DLS). The size distribution analysis showed that the Ag NPs were in the range of 19–41 nm with an average size about 27 nm (Fig. 4c). TEM image (Fig. 4b) demonstrated that the Au NPs were generally round in shape, which had a strong tendency to aggregate with each other. DLS characterization showed that the Au NPs were in the range of 11–48 nm with an average size about 23 nm (Fig. 4d). It should be noted that the TEM image and the size distribution of the Au and Ag NPs which have been stored for 100 days are quite similar to those of freshly prepared ones.

### 3.3. Other special properties of TA–PEG hydrogel

Given the facile preparation process and combined with pH responsiveness and negligible cytotoxicity, the natural polyphenol TA and Fe^III^ were chosen as the organic ligand and the inorganic cross-linker, respectively. Herein, the interaction between TA–PEG hydrogel and Fe^III^ was also studied. As characterized by UV-vis spectrum in Fig. S4 (ESI†), right from the start, the UV-vis spectrum of FeCl_3_ (0.002 M) aqueous solution shows characteristic absorption band at 300 nm. Although an amount of pyrogallol moieties participated in crosslinking process, the remaining catechol moieties belonging to TA–PEG hydrogel were also readily for metal chelation. Few minutes after the hydrogel immersed in FeCl_3_ solution, the characteristic adsorption peak of Fe^III^ at 300 nm gradually decreased along with a new adsorption peak increased at 264 nm.^35^ Meanwhile, the color of the hydrogel gradually changed to black when subjected to FeCl_3_ solution for 30 min, which indicated the typical formation of metal–phenolic complexes.

**Fig. 5:**
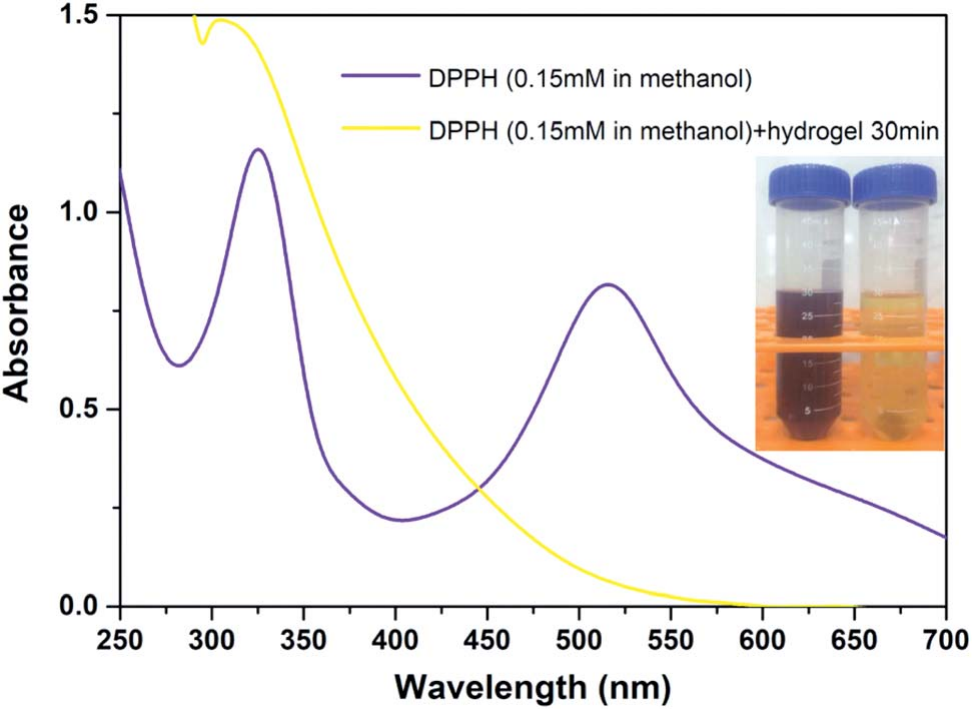
Antioxidant ability of TA–PEG hydrogel after 30 min incubation of DPPH solution (0.15 mM in methanol).

The antioxidant activity of TA–PEG hydrogel was investigated by a DPPH˙ assay. For decades, food polyphenols, including tannic acid, have aroused widespread concern owing to their roles as antioxidants, antimutagens, and scavengers of free radicals and their implication in the prevention of pathologies such as cancer and cardiovascular disease.^36^ Epidemiologic studies have shown a correlation between an increased consumption of phenolic antioxidants and a reduced risk of cardiovascular disease and certain types of cancer. Similarly, moderate consumption of red wine, which is rich in polyphenols, has been associated with a low risk of coronary heart disease.^37^ As can be seen in Fig. 5, because of a strong absorption band centered at about 520 nm, the DPPH radical has a deep violet color in solution, and it becomes colorless or pale yellow when neutralized. Overall, the antioxidant ability of the TA–PEG hydrogel was up to 94% after 30 min incubation of DPPH solution.

**Fig. 6:**
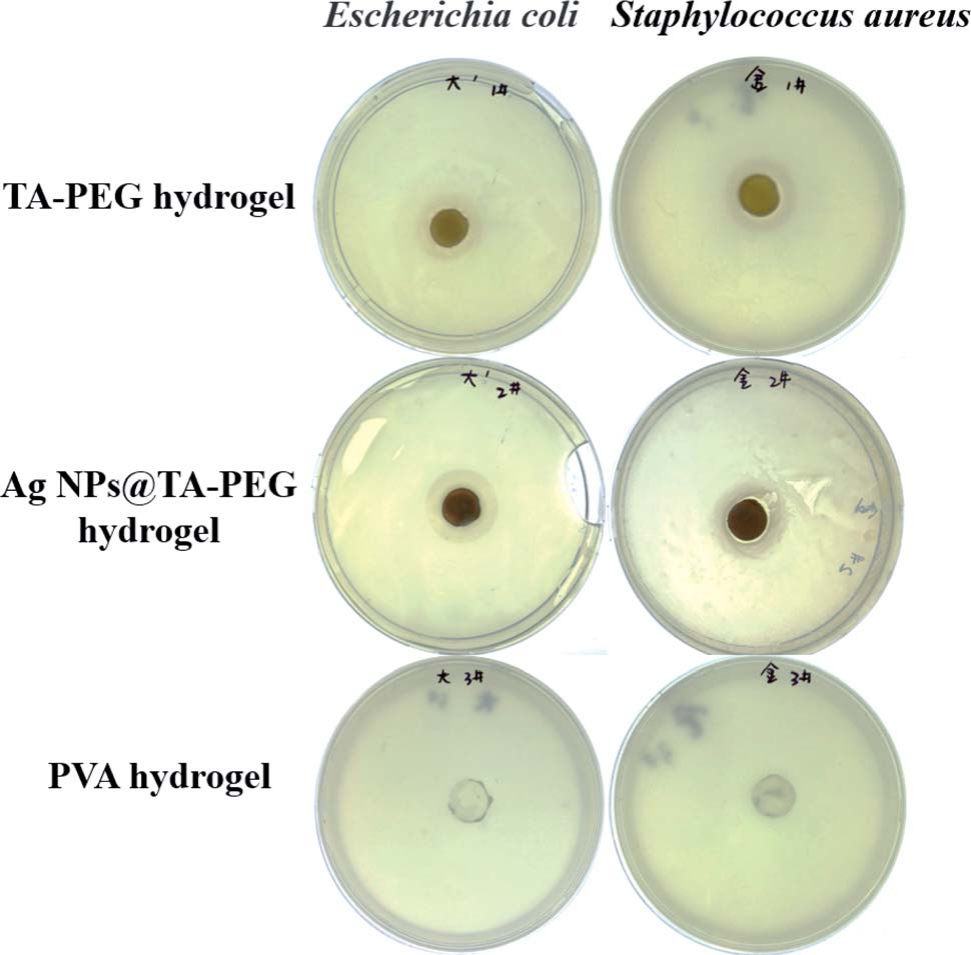
Photographs of the inhibition zones of TA–PEG hydrogels as well as Ag NPs@TA–PEG hydrogels against *E. coli* and *S. aureus*.

**Fig. 7:**
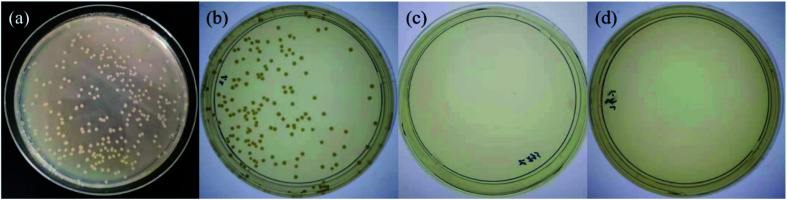
Shake flask trials against *Escherichia coli* for control group (0 h) (a), control group (18 h) (b), TA–PEG hydrogel group (c) and Ag NPs@TA–PEG hydrogel group (d).

Both TA and Ag NPs are known to possess antibacterial activities. As can be seen in Fig. 6 and Table S2 (ESI†), TA–PEG hydrogel as well as Ag NPs@TA–PEG hydrogel performed desirable antibacterial activity against *S. aureus* and *E. coli*, whereas PVA hydrogel (10 wt%) was devoid of antibacterial activity. These results revealed that the antibacterial activity of the hydrogels was due to the introduction of polyphenols or Ag NPs which are relatively safe. As can be seen in Fig. 7 and Table S2,† the results of shake flask method showed that there were many colonies in the absence of hydrogels (Fig. 7a and b), while the survival of colonies completely disappeared as TA–PEG hydrogel and Ag NPs@TA–PEG hydrogel were involved (Fig. 7c and d). In other words, both polyphenol based TA–PEG hydrogel and Ag decorated TA–PEG hydrogel were effective antibacterial agent against *E. coli*. As illustrated by Table 1, the bacteriostasis rate of these hydrogels can be >99.99%. The antimicrobial mechanisms for TA–PEG hydrogel can be summarized as follows: (i) the astringent property of tannic acid may introduce complexation with enzymes or substrates. Many microbial enzymes in raw culture filtrates or in purified forms are inhibited when mixed with tannin residues within the TA–PEG hydrogel. (ii) A tannin's toxicity may be related to its action on the membranes of microorganisms. (iii) Complexation of metal ions by pyrogallol and catechol moieties may account for tannin toxicity.^38^ Moreover, the antibacterial mechanisms for Ag NPs@TA–PEG hydrogel relates to a very large specific surface area for the release of ionic silver derived from nanocrystalline silver, and even a small amount of silver provides a bactericidal action.^39^ Therefore, it is expected that TA–PEG hydrogel and Ag NPs@TA–PEG hydrogel are promising in the field of wound dressing. Though could not heal itself once cut into pieces, TA–PEG hydrogel showed adhesive properties owing to inherent polyphenol groups from TA. As can be seen in Fig. S5 (ESI†), two pieces of the hydrogel stuck together after 5 min sectional interaction. Further works focused on enhancing mechanical properties will be initiated to fulfill practical use.

**Table 1:** Shake flask antibacterial test results of TA–PEG hydrogel and Ag NPs@TA–PEG hydrogel against *Escherichia coli*

Sample	Diluent factor	Colony count	Concentration of the total bacteria colonies (CFU mL^−1^)	Bacteriostasis (%)
Control group (0 h)	10^5^	270	2.70 × 10^7^	—
Control group (18 h)	1000	139	1.39 × 10^5^	—
TA–PEG hydrogel group	1000	0	0	>99.99
Ag NPs@TA–PEG hydrogel group	1000	0	0	>99.99

## 4. Conclusion

To sum up, a novel TA based hydrogel was synthesized *via* Mitsunobu polymerization method, in which TA worked as an acidic pronucleophile and PEG worked as a macromolecular substrate. This route is facile, robust, metal free and moldable under mild condition. The formation of TA–PEG polymeric network was confirmed by the results from FI-IR spectrum and dynamic rheology study. Took the polyphenol nature of TA into consideration, the resulted TA–PEG hydrogel exhibited special properties such as antioxidant ability, metal chelation ability, degradability and antibacterial activity. Moreover, *in situ* formation of Au or Ag NPs could be carried out *via* direct soaking of the hydrogel into HAuCl_4_ or AgNO_3_ aqueous solution without external reductants, probably due to the interactions between polyphenol units and noble metal ions. Thus, it was concluded that TA–PEG hydrogel has great clinical potential in fields such as wound healing, tissue engineering and gel preparation.

## Conflicts of interest

There are no conflicts to declare.

## Supplementary Material

RA-010-C9RA09229C-s001

RA-010-C9RA09229C-s002

RA-010-C9RA09229C-s003
