# Supplementary material for: Synthesis and characterization of tannic acid–PEG hydrogel via Mitsunobu polymerization
Source: RSC Adv. 2020 Jan 9;10(3):1724–32. doi: 10.1039/c9ra09229c (PMC9048105; doi:10.1039/c9ra09229c)
Supplement: RA-010-C9RA09229C-s003 [file RA-010-C9RA09229C-s003.pdf]

Electronic Supplementary Material (ESI) for RSC Advances.
This journal is © The Royal Society of Chemistry 2019

Supporting Information to "Synthesis and Characterization of Tannic Acid-PEG Hydrogel via Mitsunobu Polymerization
"

Chen Chen, [1] **Xi-wen Geng,** [2] **Ya-hui Pan,** [1] **Yu-ning Ma,***[,1] **Yu-xia Ma,***[,1] **Shu-zhong Gao**[1]**, and Xiao-jun Huang**[3]

[1]Key Laboratory of New Material Research Institute, Department of Acupuncture-Moxibustion and Tuina, Shandong University of Traditional Chinese Medicine, Jinan 250355, China

[2]Key Laboratory of Stem Cell and Translational TCM, Experimental center, Shandong University of Traditional Chinese Medicine, Jinan 250355, China

[3]MOE Key Laboratory of Macromolecular Synthesis and Functionalization, Department of

Polymer Science and Engineering, Zhejiang University, Hangzhou 310027, China

E-mail: myning0405@163.com; myxia1976@163.com

Table S1 Synthesis of TA-PEG hydrogel: Reagent ratios, ratio of functional groups and results on whether the sample gelled or not.

Sample	TA (mmol)	PEG 6000 (mmol)	Pyrogallol groups/hydroxyl groups	Gelation
TA-PEG	0.100	0.250	1.00	Y
TA-PEG-1	0.100	0.500	0.50	N
TA-PEG-2	0.100	0.125	2.00	N

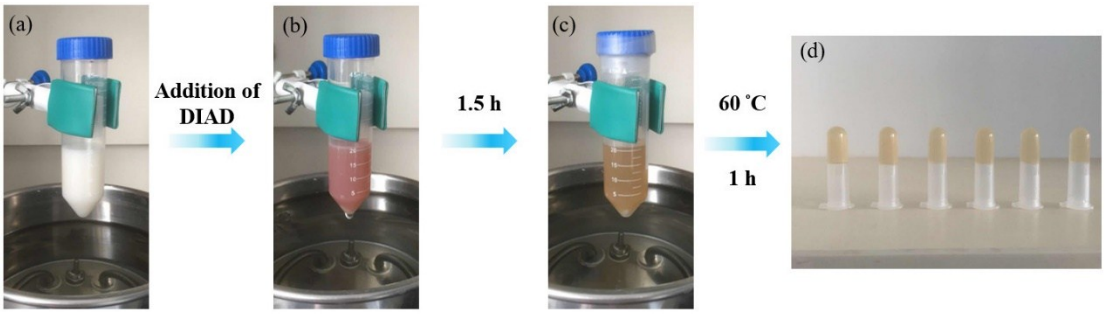

Figure S1. The photo pictures of the reaction phenomena for the synthesis of TA-PEG hydrogel. (a) The heterogeneous solution of TA, PEG6000 and PPh_3 in CH_3CN; (b) Orange-coloured solution obtained at the end of DIAD titration at

0 °C; (c) The homogeneous solution after constant stirring for 1.5 h; (d) TA-PEG hydrogels.

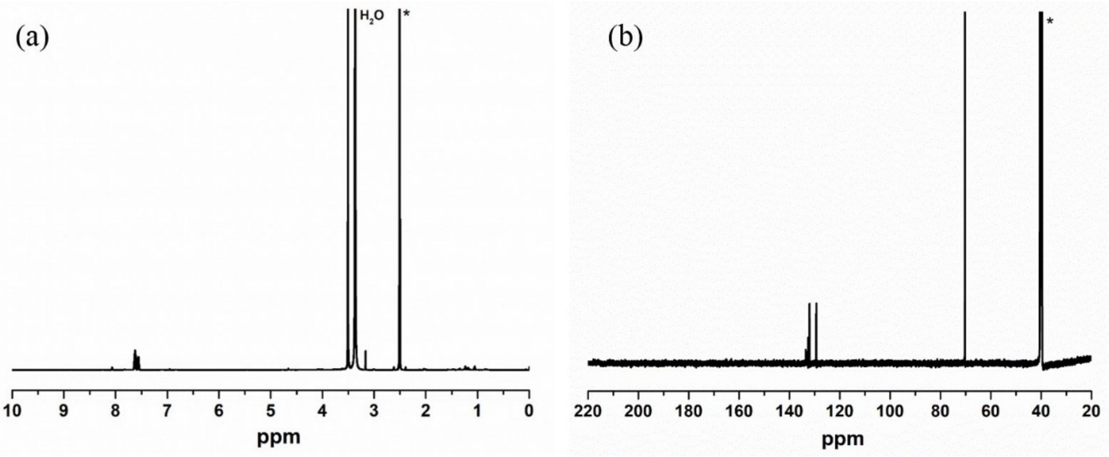

Figure S2 ^{1}H NMR (a) and ^{13}C NMR (b) results of the hydrolyzed TA-PEG hydrogel after 10 days in 10 mM PBS (pH 7.4) at 37 °C.

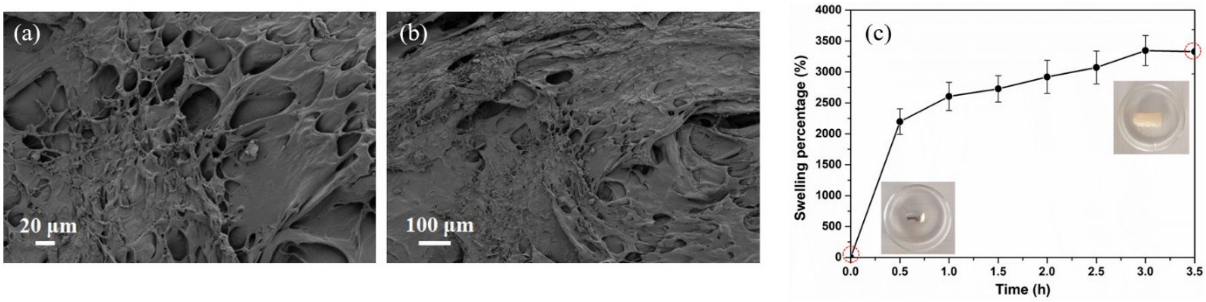

Figure S3. Morphology and swelling properties of TA-PEG hydrogel. (a) and (b) Typical FE-SEM micrographs of TA-PEG hydrogel; (c) Swelling percentage of TA-PEG hydrogel.

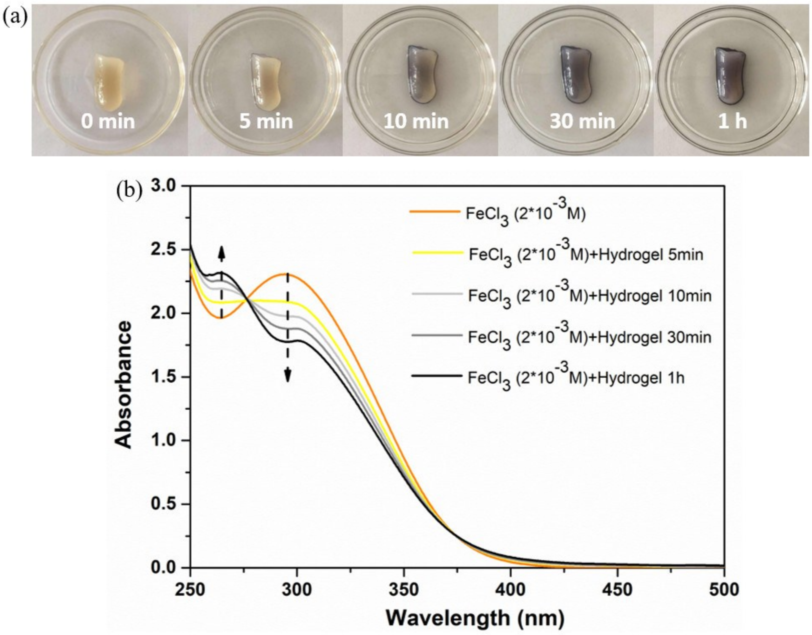

Figure S4. The metal-phenol complexion between Fe^{III} iron and TA-PEG hydrogel. (a) Photographs of TA-PEG hydrogel in treatment of $FeCl_3$ (0.002 M); (b) In-situ formation of metal-phenol complexion objected by UV-vis spectrum.

Table S2. Antibacterial activity data of TA-PEG hydrogel against *E. coli* and *S. aureus* (*P<0.05, #P<0.1).

**Sample name**	**Inhibition zone(mm)**	
	E. coli	***S. aureus***
**TA-PEG hydrogel**	**18.8***	**21.7#**
**Ag NPs@TA-PEG hydrogel**	**19.2#**	**20.5#**
**PVA hydrogel**	**0**	**0**

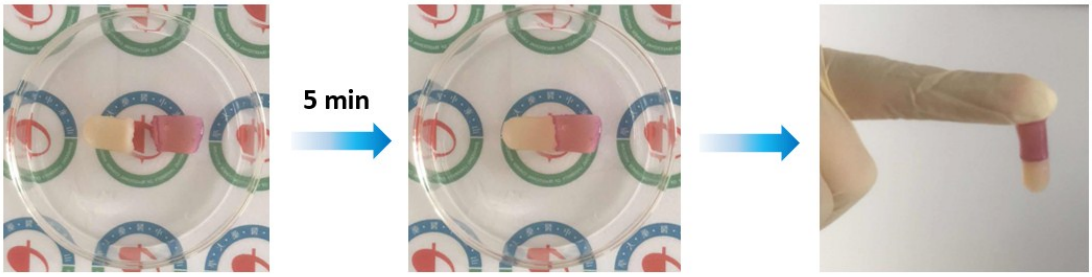

Figure S5. Adhesive property of TA-PEG hydrogel (only one piece of the hydrogel was stained by *Eosin Y*).

Supporting Movies

Movie S1: In-situ formation of Ag NPs. The TA-PEG hydrogel were cut into pieces (1 cm in diameter and 3mm in height) and the concentration of $AgNO_3$ was 0.1 wt%. The movie is real-time.

Movie S2: In-situ formation of Au NPs. The TA-PEG hydrogel were cut into pieces (1 cm in diameter and 3mm in height) and the concentration of $HAuCl_4$ was 0.01 wt%. The movie is real-time.
